# The Respiration of Plants

**Published:** 1895-09-14

**Authors:** 


### The Respiration of Plants.

In *Science Progress* there is an interesting article by R. Frost Blackman, M.A., Demonstrator of Botany in the University of Cambridge, in which he shows the importance of the stomata which occur on the leaves of plants in the performance of their respiratory functions. It has long been recognised that both watery vapour and carbonic acid are transfused with much greater freedom by way of the stomata, which form the openings of an extensive system of intercellular spaces existing in the leaves, especially in their lower strata, than through the epidermis, and that these stomata and spaces were largely concerned in the lung-like function of the leaves, by means of which, as in animals, oxygen is absorbed and carbonic acid excreted by the living plant; but as to the route of that large intake of carbonic acid which occurs during active assimilation, experiments appeared to point to the conclusion that it proceeded also, if not chiefly, through the portion of the cuticle which was free from stomata, the critical experimental proof of this being that put forward by Boussingault, which consisted in showing that of two otherwise similar leaves, the one which had its stomata mechanically occluded did not decompose any less carbon dioxide than the leaf with its stomata in open functional condition. The probability that the respiratory and the assimilative interchange of gases took place by different routes was also made probable by experiments, which seemed to show that leaves exhaled carbonic acid even in bright sunshine, when they were known to be at the same time actively absorbing it. Mr. Blackman, however, is able to show by a series of very careful experiments that the stomata are practically the sole path both for the exhalation of respiratory carbonic acid and also for the absorption of the carbonic acid required by the plant in its periods of active assimilation. Not only does he do this, but by other experiments he is able to explain away the apparently opposite results obtained by earlier investigators. Like many other contributions to *Science Progress*, this article is not only a careful record of important scientific investigations, but is an interesting *résumé* of the present position of knowledge on the subject treated of.

